# Superior semicircular canal dehiscence with concomitant otosclerosis—A literature review and case discussion

**DOI:** 10.1002/ccr3.1822

**Published:** 2018-10-24

**Authors:** Omer J. Ungar, Ophir Handzel, Oren Cavel, Yahav Oron

**Affiliations:** ^1^ Department of Otolaryngology Head and Neck Surgery and Maxillofacial Surgery Tel Aviv Sourasky Medical Center Sackler School of Medicine Tel Aviv University Tel Aviv Israel

**Keywords:** middle ear surgery, otosclerosis, stapes fixation, stapes surgery, superior semicircular canal dehiscence

## Abstract

Computed tomography scan should be performed as a routine before every stapes surgery, in order to exclude concomitant superior semicircular canal dehiscence, since no other clinical, audiological, or electro‐physiological criteria are available to exclude concomitant superior semicircular canal dehiscence in the otosclerotic temporal bone.

## 1 INTRODUCTION

Superior semicircular canal dehiscence and otosclerosis are two distinctive pathological conditions of the otic capsule, with overlapping clinical presentation. The otosclerotic oval window occlusion can mask the presence of a “third window” by setting the inner ear back to “two windowed osseous labyrinth” status. Stapes surgery in this setting will unmask the symptoms of the latent Superior semicircular canal dehiscence. The aim of this review was to present the clinical presentation and post‐operative outcomes of this patient population. A search for all English language articles in “MEDLINE” via “PubMed” and “Google Scholar” was conducted. In addition to that, a demonstrative case of otosclerosis and superior semicircular canal dehiscence is described. Seven articles, describing 14 patients with 16 ears, were included. The median age of the patients was 46.5 years. Conductive hearing loss and tinnitus were the most common symptoms at presentation. Eight patients were operated, and the diagnosis of concomitant Superior semicircular canal dehiscence was made postoperatively in six patients. The most common post‐operative complaint was the absence of hearing gain. The diagnosis of Superior semicircular canal dehiscence should be kept in mind when treating a patient with conductive hearing loss. In a case with a high level of suspicion of Superior semicircular canal dehiscence, this diagnosis should be ruled out pre‐operatively.

Superior semicircular canal dehiscence and otosclerosis are two distinctive pathological conditions involving the otic capsule. Superior semicircular canal dehiscence is defined by loss of the tegmen over the superior semicircular canal, producing potential third bony window of the inner ear. Otosclerosis is a metabolic disease of the otic capsule, resulting in progressive stapes fixation to the oval window. This process reduces the inner ear to one mobile window only. Both entities affect the transfer of acoustic energy in the inner ear, with overlapping clinical presentations that may cause difficulty in distinguishing between the two.^1^, ^2^, ^3^, ^4^, ^5^ In patients presenting with conductive hearing loss, superior semicircular canal dehiscence is best ruled out before exploring the middle ear in search of correctable otosclerosis. In this situation, only one pathology may exist. However, they may co‐exist. It is believed that the acquired otosclerotic oval window occlusion can mask the presence of a “third inner ear window” by eliminating the function of the oval window. Symptoms of superior semicircular canal dehiscence maybe unmasked by surgery to remove fixation of the stapes^6^, ^7^, ^8^, ^9^ reflected by lack of or incomplete hearing improvement, the appearance of vestibular symptoms, or combination of both.^8^, ^10^

Because of the rarity of symptomatic superior semicircular canal dehiscence and the fact only about half of the otosclerotic ears undergo stapes operation,^11^, ^12^, ^13^ the combination of otosclerosis with post‐stapedectomy apparent superior semicircular canal dehiscence is rare. As a result, publications of clinical cases are restricted to several case reports and small series, summed up to 12 patients (13 ears).^4^, ^6^, ^7^, ^8^, ^9^, ^14^, ^15^ This is a review of the clinical presentation and post‐operative outcomes of patients with superior semicircular canal dehiscence after stapes surgery. Clinical example of the dilemmas is presented.

## 2 MATERIALS AND METHODS

A search for all English language articles in “MEDLINE” via “PubMed” and “Google Scholar” was conducted. The publication date chosen was from March 1998, when superior semicircular canal dehiscence was first described 16 to April 2018. The search included the following terms and Boolean operators: [otosclerosis] OR ([stapes] and [surgery]) AND ([superior] and [canal]). Included are all articles which presented at least one individual case report, describing a patient with otosclerosis and concomitant superior semicircular canal dehiscence. Articles that did not describe the pre‐operative clinical presentation, or the post‐operative outcomes (when performed) in sufficient detail, were used for demographic analysis only. If a surgery was performed in order to restore stapes mobility, the post‐operative outcome was reviewed.

Each clinical case was analyzed in terms of demographic data, clinical presentation, surgical findings, and post‐surgical outcome, as well as radiological findings. Age in this review refers to the age at the time the primary operation was performed, or the age when the diagnosis of superior semicircular canal dehiscence was made, the earlier one.

### 2.1 Ethical considerations

This study reviews published data, and add one case presentation without identification details. The study was approved by the institutional ethics committee.

Categorical variables were described as frequency and percentage. Continuous variables were described as median and interquartile range. Continuous variables were evaluated for normal distribution using a histogram and a Q‐Q plot, and described as median and range. All the statistical analyses were 2‐tailed. A *P* value <0.05 was considered statistically significant. SPSS (IBM SPSS Statistics for Windows, Version 22.0. Armonk, NY: IBM Corp.) was used for all the statistical analyses.

## 3 RESULTS

### 3.1 Case presentation

**Figure 1:**
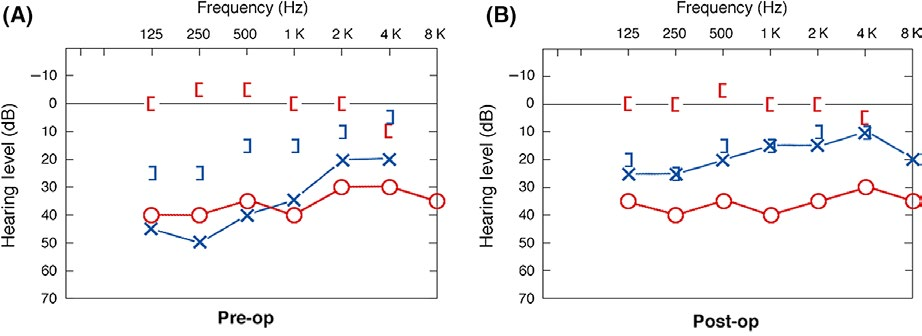
Pure tone audiogram of the patient described in the case presentation, at presentation (A) and post‐operatively (B)

**Figure 2:**
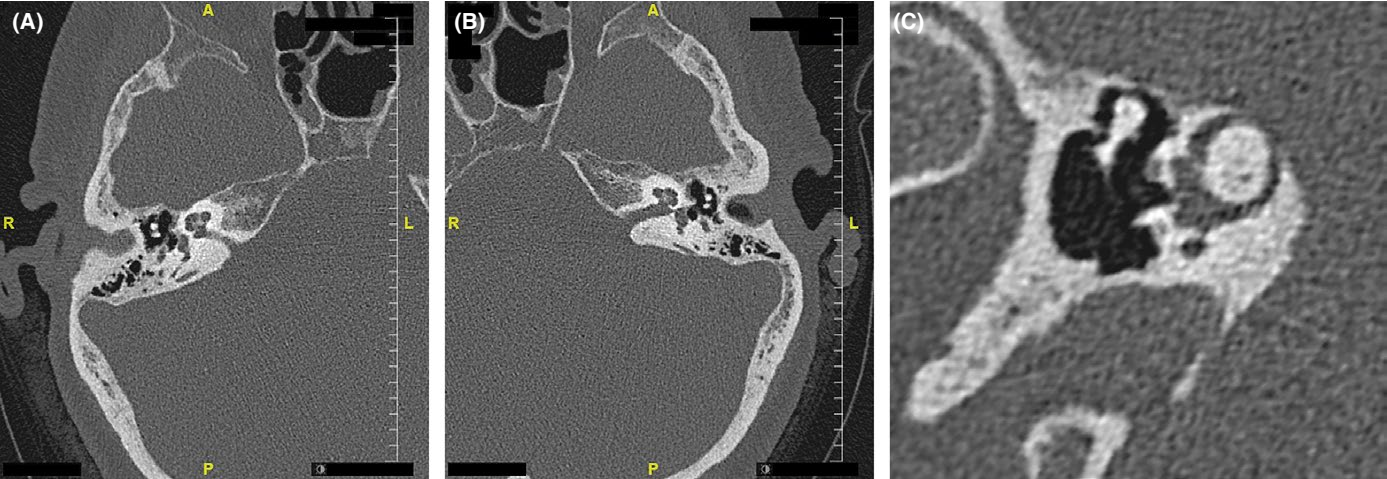
An axial right (A) and left (B) computed tomography scan of the patient described in the case presentation. Bilateral otosclerosis is seen. A reconstructed Pöschl's view of the right temporal bone (C). A dehiscence of the right superior semicircular canal is easily seen

A 65‐year‐old woman presented with a complaint of progressive hearing loss without dizziness or vertigo. Hearing loss was of mixed conductive and sensorineural (Figure 1A). Physical examination was normal, without nystagmus or eye deviation, with the exception of negative Rinne tuning fork (512 Hz) testing. Computed tomography scan (Figure 2) depicted bilateral otosclrosis (Figure 2A,B) and right‐sided superior semicircular canal dehiscence (Figure 2C). A successful left‐sided stapedectomy was performed, confirming the diagnosis of otoscelrosis and closing the air‐bone gap to less than 10 dB (Figure 1B). Six months after the procedure, the patient expressed her wish to have the same surgery on the right but was advised to use a hearing aid instead. Electro‐physiological assessment (vestibular evoked myogenic potentials, video nystagmography, and video head impulse test) was not performed since the patient decided to avoid surgery on the right side. The total follow‐up duration was 2 years.

### 3.2 Literature review

**Figure 3:**
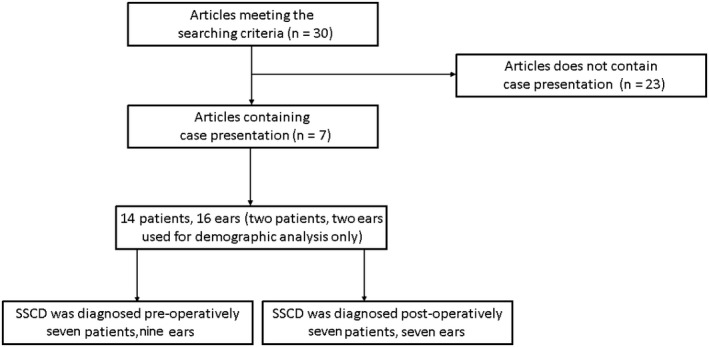
Inclusion and exclusion process of the enrolled publications of this review

Thirty articles met the above‐mentioned searching criteria. Twenty‐three papers did not include any case presentation and were excluded, leaving seven articles,^4^, ^6^, ^7^, ^8^, ^9^, ^14^, ^15^ describing 14 patients with 16 ears, for statistical analysis. Cases 5 and 6 (two patients, two ears)^9^ were used for demographic analysis only, due to insufficient clinical presentation and post‐operative outcome (Figure 3), leaving 12 patients with sufficient data.

The median age of the patients was 46.5 years, (range: 11‐67). Eight (57%) patients were males and 6 (43%) were females. The prevalence in terms of laterality was equal: eight right ears and eight left ears were affected, meaning that 12 patients were affected unilaterally and two patients were affected bilaterally. Both bilaterally affected patients were females.

Six patients (eight ears) were diagnosed with otosclerosis and concomitant superior semicircular canal dehiscence before surgery, meaning that both patients with bilateral disease were diagnosed pre‐operatively. Eight patients were diagnosed with concomitant superior semicircular canal dehiscence post‐operatively. There was no significant gender or age distribution among the pre‐ and post‐operative diagnosis of concomitant Superior semicircular canal dehiscence (Table 1).

**Table 1:** List of the enrolled publications of this review

Reference	Case #	Gender	Age	Side	Diagnosis of SSCD	Available Audiogram?	HL	Tinitus	Hennebert's sign	VEMP	Primary surgery	Revision surgery
7	1	M	30	L	Post‐op.	N	CHL	NA	NA	Reduced	Stapedotomy	0
6	2	M	54	L	Post‐op.	N	MHL	NA	NA	Reduced	Stapedectomy	2
5	3	F	48	L	Post‐op.	N	NA	NA	NA	NA	Stapedectomy	2
13	4	F	33	B	Pre‐op.	Y	MHL	Y	(‐)	Reduced	Stapedotomy	0
8	5	M	67	R	Post‐op.	N	NA	NA	NA	NA	NA	NA
8	6	F	11	L	Post‐op.	N	NA	NA	NA	NA	NA	NA
14	7	F	37	B	Pre‐op.	Y	MHL	Y	(‐)	Reduced	Not operated	
14	8	M	45	R	Pre‐op.	Y	No	Y	NA	Normal	Stapedotomy	0
14	9	F	49	R	Pre‐op.	Y	CHL	Y	NA	Absent	Not operated	
3	10	F	27	R	Post‐op.	Y	CHL	N	NA	Reduced	Stapedectomy	0
3	11	M	52	L	Post‐op.	Y	MHL	N	NA	Reduced	Stapedectomy	0
3	12	M	49	R	Post‐op.	Y	CHL	N	NA	Reduced	Stapedectomy	1
3	13	M	45	L	Pre‐op.	Y	CHL	N	NA	Reduced	Not operated	
3	14	M	59	R	Pre‐op.	Y	CHL	N	NA	NA	Not operated	

SSCD, superior semicircular canal dehiscence; M, male; F, Female; HL, hearing loss; CHL, conductive HL; MHL, mixed HL; N, no; Y, yes; NA, not available; VEMP, vestibular evoked myogenic potential.

**Figure 4:**
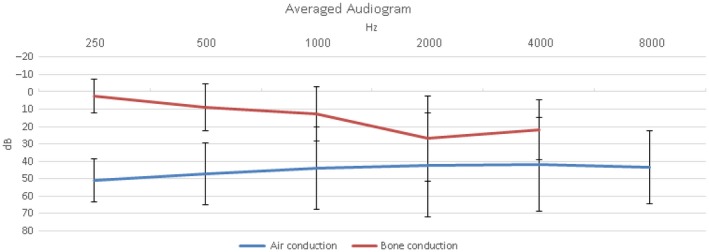
Averaged hearing thresholds on presentation, of the enrolled case presentations

The most prevalent pre‐operative presentation was hearing loss, described by 11 of 12 patients. The most prevalent hearing loss type was conductive hearing loss (six patients), followed by mixed hearing loss and non‐specified hearing loss (four and one patients, respectively). Nine reports added formal audiograms from the initial presentation (Figure 4). One patient reported of hyperacusis (case 8), and two patients described autophony (case 7, 8). One of those patients presented with long‐term hyperacusis to bone conduction (hearing his own joints and pulse) that resolved spontaneously as air conduction hearing loss developed (case 2).

The second most prevalent pre‐operative symptom was tinnitus (4 of 12 patients). The absence of tinnitus was reported in five patients, and there was no reference to the presence or absence of tinnitus in three case reports (Table 1). Both bilateral cases had tinnitus. Aural fullness was presented in one patient (case 9).

Sound and pressure evoked dizziness were documented as an initial presentation only in two patients (cases 3 and 8, respectively). In seven patients, it was reported to be absent. Loss of balance was documented in one patient (case 2).

The physical examination was either normal or not reported at all, except the expected lateralization of 512 Hz tuning fork and negative Rinne in the otosclerotic ear. Hennebert's sign was reported to be negative in two patients (cases 4 and 7). These patients had bilateral otosclerosis with concomitant superior semicircular canal dehiscence, and their diagnosis was made intra‐operatively. In 10 patients, the result of Hennebert's sign was either not reported or not assessed.

The primary operation was stapedotomy or stapedectomy in five and three patients, respectively. Among them, in two and six patients, the diagnosis of concomitant superior semicircular canal dehiscence was made pre‐operatively (25%) and post‐operatively (75%), respectively. The suspicion of concomitant superior semicircular canal dehiscence was raised after the absence of hearing improvement and/or vestibular symptoms developed in five and three patients, respectively. Four patients did not undergo surgical treatment (cases 7, 9, 13, 14) because the medical team assumed that the surgery would result with no hearing gain due to the 3rd window. Three patients underwent five revision surgeries, due to the absence of hearing loss improvement (two patients) and/or vestibular symptoms (two patients; Table 1). No revisions were indicated when a pre‐operative diagnosis of concomitant superior semicircular canal dehiscence was made.

It is important to emphasize one case report,^4^ presenting a patient who underwent explorative tympanotomy due to clinical diagnosis of otosclerosis. Because of good ossicular chain mobilization, no surgical manipulation was performed. Post‐operative computed tomography showed an 8 mm superior semicircular canal dehiscence. This patient was not included in the statistical analysis due to negative diagnosis of otosclerosis. One patient (case 1) underwent a post‐stapedotomy superior semicircular canal dehiscence correction via temporal craniotomy approach.

Various neuro‐physiological tests were performed as soon as a concomitant superior semicircular canal dehiscence was suspected, either pre‐ or post‐operatively. The most sensitive was vestibular evoked myogenic potential test, which was conducted in 10 patients (six cervicals and four occulars) and found to be suggestive of concomitant superior semicircular canal dehiscence in 8 (80%) patients. Two patients had absent and normal vestibular evoked myogenic potentials (cases 9 and 8, respectively; Table 1).

## 4 DISCUSSION

The actual prevalence of otosclerosis with concomitant ipsilateral superior semicircular canal dehiscence is unknown. It was reported to be as high as 5.3%,^17^ but clinical practice and temporal bone surveys suggest a much lower prevalence. Despite the fact that these two conditions are a result of an otic capsule pathology, the pathogenesis pathway is very different for each of the diseases.

The prevalence of otosclerosis differs as a function of the diagnostic method. Clinically, otosclerosis affects about 1% of the general population, as calculated by retrospective cohorts,^13^, ^18^ and even archeological material.^19^Histologically, this prevalence is as high as 2.5%.^20^ As experience with superior semicircular canal dehiscence accrues, it is emerging that it is not such a rare condition. Histopathological studies on temporal bones suggest superior semicircular canal dehiscence prevalence of 0.5%, and the incidence of a very thin tegmen (also called “near dehiscent”) over the superior semicircular canal (<0.1 mm) to be 1.5%.^21^, ^22^ Radiologically, the prevalence of dehiscent‐appearing superior semicircular canal on thin‐section TB scanning is 4‐6%, a value that is much higher than anticipated by pathologic studies.^23^, ^24^ The prevalence of radiographic superior semicircular canal dehiscence is higher in ears with chronic otitis media.^25^Whether radiographic superior semicircular canal dehiscence is more common in the setting of increased intracranial pressure remain debatable.^26^

According to the prevalence of each of the diseases, the concomitant combination is calculated to be in the range of 150/100 000 of the general population, and probably much higher among revision stapes surgery candidates.

Most of the radiographically diagnosed superior semicircular canal dehiscence are asymptomatic, due to a tegmen thickness which is thinner than the resolution limit of the radiographic modality used, even when high‐resolution computed tomography is used. Such a thin bone might appear dehiscent on computerized tomography of the temporal bone due to voxel intensity averaging.^27^, ^28^ In order to minimize this bias, images should subsequently be reformatted to include views in the plane of the superior semicircular canal (Pöschl's view) and perpendicular to this plane (Stenver's view) 23, 29, 30 The radiological diagnosis of probable superior semicircular canal dehiscence is retained only when the dehiscence is apparent in both reconstruction series. It should be kept in mind that a radiographically diagnosed superior semicircular canal dehiscence does not necessarily lead to an active third inner ear window.^31^, ^32^ A real superior semicircular canal dehiscence may remain asymptomatic; conceivably, the brain itself functionally plugs the dehiscent canal, especially in overweight patients with elevated intracranial pressure.

The diagnosis of superior semicircular canal dehiscence should be raised when a patient presents with a low‐frequency conductive hearing loss and bone conduction thresholds that are better than 0 dB. The conductive hearing loss is the result of dissipation of accoustic energy through the perilymphatic compartment against the dure, attenuating the pressure wave to the cochlea. In such a case, performing a temporal bone computed tomography with the above‐mentioned views, and a vestibular evoked myogenic potential examination, in which lower than normal thresholds are expected.^4^ However, when an inner ear is influenced by both otosclerosis and superior semicircular canal dehiscence proving functionality of the 3rd window can be difficult or impossible. The conductive component of the hearing loss may be ascribed to either condition although supra‐normal bone conduction thresholds can be caused by superior semicircular canal dehiscence but not otoscelerosis. Similarly, vestibular evoked myogenic potential results can be difficult to interpret. Both air and bone stimulation can yield unpredictable stimulation of the 3rd window, as due to the effect of otosclerosis they are often asymmetrical. Otoacustic emissions are likely to be absent.

Performing stapes surgery in an ear with superior semicircular canal dehiscence has a number of potentials risks. First, it is possible, although yet to be proved, that surgery may turn a non‐active 3rd inner ear window into an active one. Sound energy is attenuated by the fixed stapes, and hence, the drive for auditory symptoms is smaller. It may also change the mechanism of energy dissipation in the inner ear preventing vestibular symptoms. Second, in the face of a 3rd window, the inner ear may have lower resistance to sound penetrance. As fenestration or removal of the stapedial footplate transfer some abnormal energy to the inner ear, it could increase the higher risk of inner ear damage during operation opening the stapes footplate. The same may apply to other rarer 3rd window such as a dehiscent of the other semicircular canals or an enlarged vestibular aqueduct. The risk may exist in other procedures mobilizing the ossicular chain such as tympanoplasty or ossiculoplasty. Based on these potential risks, the patient in the case presentation was advised to avoid surgery and to use behind the ear hearing aid, as a first‐line treatment.

## 5 CONCLUSIONS

The diagnosis of superior semicircular canal dehiscence should be kept in mind when treating a patient with conductive hearing loss. In a case with a high level of suspicion of superior semicircular canal dehiscence, this diagnosis should be ruled out pre‐operatively. The importance of pre‐operative diagnosis of concomitant superior semicircular canal dehiscence in the presence of otosclerosis is dual: Pre‐operative, the surgeon and the patient should be aware that the hearing loss might not improve as a result of a stapes surgery and that there might be an increased risk of inner ear complications. Second, a revision surgery may be prevented in the absence of post‐operative improvement. The possible coexistence of ipsilateral otosclerosis and superior semicircular canal dehiscence supports ordering high‐quality computed tomography scans before every stapes surgery, since no other clinical, audiological, or electro‐physiological criteria are available to exclude concomitant superior semicircular canal dehiscence in the otosclerotic temporal bone.

## AUTHOR CONTRIBUTION

OJU: involved in manuscript writing. OC: involved in literature review. OH: prepared case report. YO: involved in manuscript revision.

## CONFLICT OF INTEREST

None declared.
